# Social nudges for vaccination: How communicating herd behaviour influences vaccination intentions

**DOI:** 10.1111/bjhp.12556

**Published:** 2021-09-08

**Authors:** Aleksandra Lazić, Kalina Nikolova Kalinova, Jali Packer, Riinu Pae, Marija B. Petrović, Dora Popović, D. Elisabeth C. Sievert, Natalie Stafford‐Johnson

**Affiliations:** ^1^ University of Belgrade Serbia; ^2^ Sofia Bulgaria; ^3^ King's College London UK; ^4^ University College London UK; ^5^ University of Zagreb Croatia; ^6^ Institute of Social Sciences Ivo Pilar Croatia; ^7^ Hamburg University of Applied Sciences Germany; ^8^ Dublin Ireland

**Keywords:** experiment, health communication, herd immunity, immunisation, registered report, social norms, vaccination intention

## Abstract

**Objectives:**

This Registered Report attempted to conceptually replicate the finding that communicating herd immunity increases vaccination intentions (Betsch, et al., 2017, *Nat. Hum. Behav*., 0056). An additional objective was to explore the roles of descriptive social norms (vaccination behaviour of others) and the herd‐immunity threshold (coverage needed to stop disease transmission).

**Design:**

An online experiment with a 2 (herd‐immunity explanation: present vs. absent) × 3 (descriptive norm: high vs. low vs. absent) × 2 (herd‐immunity threshold: present vs. absent) between‐subjects fractional design.

**Methods:**

Sample consisted of 543 people (aged 18–64) residing in the United Kingdom. Participants first received an explanation of herd immunity emphasising social benefits (protecting others) in both textual and animated‐infographic form. Next, they were faced with fictitious information about the disease, the vaccine, their country’s vaccination coverage (80% or 20%), and the herd‐immunity threshold (90%). Vaccination intention was self‐rated.

**Results:**

Compared to the control, communicating social benefits of herd immunity was effective in increasing vaccination intentions (*F*(1,541) = 6.97, *p* = .009, Partial Eta‐Squared = 0.013). Communicating the descriptive norm or the herd‐immunity threshold alongside the herd‐immunity explanation demonstrated no observable effect.

**Conclusion:**

Communicating social benefits of herd immunity increased self‐reported vaccination intentions against a fictitious disease, replicating previous findings. Although this result is positive, the practical relevance may be limited. Further research into the effect of social nudges to motivate vaccination is required, particularly with respect to the recent pandemic context and varying levels of vaccine hesitancy.

**Statement of contribution**

***What is already known on this subject?***

- Communicating social benefits of herd immunity sometimes increased vaccination intentions.
- Many correlational studies have linked descriptive norms to individual vaccination decisions.
- It is not yet clear whether setting collective goals influences individual vaccination decisions.

***What does this study add?***

- Tested the effect of communicating herd immunity in combined textual and animated‐infographic form.
- Replicated the finding that social‐benefit appeals increase vaccination intentions.
- Presenting descriptive norms and the herd‐immunity threshold alongside herd immunity had no effect.

## Background

Vaccination is the most effective way to protect both individuals and communities from infectious diseases. The World Health Organization (WHO, n.d.‐a) estimates that vaccination currently prevents between two and three million deaths every year. However, a growing number of people are delaying or refusing to get vaccinated, even in the absence of structural barriers (e.g., problematic access to healthcare, vaccination costs) (WHO, n.d.‐b). This has led to recent outbreaks of previously eliminated diseases, making vaccine hesitancy a major threat to global health (WHO, n.d.‐b). In 2019, for example, the United Kingdom lost its ‘measles‐free’ status, with 991 confirmed cases in England and Wales in 2018, compared with 284 cases the year before (Public Health England, 2019).

To tackle vaccine hesitancy, this study explored intervention strategies that harness social processes to motivate vaccination. More specifically, we focussed on the following three social nudges: the communication of herd immunity, the herd‐immunity threshold, and descriptive social norms.

### Herd‐immunity communication

The more people in a community that are vaccinated against a disease, the less probable it is for the disease to spread. This effect of herd immunity protects everyone but is especially important for vulnerable populations who cannot get vaccinated (such as people with serious allergies or those with weakened immune systems; Fine, Eames, & Heymann, 2011). Recent studies have shown that communicating herd immunity has the potential to increase vaccination intentions (e.g., Betsch & Böhm, 2018; Betsch, Böhm, Korn, & Holtmann, 2017; Logan et al., 2018). Specifically, communicating the social benefit (protecting others) and visually demonstrating this effect seems to have the largest impact (see also Hakim et al., 2019).

The main goal of this study was to attempt to conceptually replicate the finding that communicating the concept of herd immunity increases the willingness to get vaccinated (Betsch et al., 2017). The original study by Betsch et al. (2017) was conducted as an online experiment, with a non‐representative sample of 2,107 adult participants from seven countries (the United States, the Netherlands, Germany, India, Hong Kong, Vietnam, and South Korea). The present replication study was also conducted as an online experiment, but with a sample of participants who live in the United Kingdom.

Given that herd immunity is under‐explained and under‐utilised in vaccine advocacy (Brockmann, 2017), it is important to test if the effect of communicating herd immunity replicates. It is especially relevant to see whether this effect is stable across countries with varying vaccination laws and levels of anti‐vaccination sentiment. Furthermore, our replication study may have practical implications for the design of herd‐immunity communication. The original study used an interactive simulation. As an alternative to this, we used an animated infographic. This medium may be easier to disseminate on television and social networks and may be more familiar to participants.

Like the original study, we explored decision‐making about a hypothetical disease transmitted directly through contact with an infected person or indirectly by touching contaminated objects. The effect of herd‐immunity communication may be dependent on the mode of disease transmission. For example, in the case of sexually transmitted infections (STIs), this could be due to the extreme heterogeneity in the risk of acquiring and transmitting STIs or the fact that STIs affect sexually active people (Garnett, 2005).

Hypothesis 1 Participants who learn about the social benefit of herd immunity visualised by an animated infographic will show higher vaccination intentions compared to participants who do not learn about it.

### Descriptive norm communication

Descriptive norms (i.e., what most others are doing) can be a powerful source of informational social influence. By signalling what will likely be an effective and reasonable course of action under the given circumstances (Cialdini et al., 2006; Cialdini, Reno, & Kallgren, 1990), descriptive norms might also motivate individual vaccination decision‐making.

According to a review by Brewer, Chapman, Rothman, Leask, and Kempe (2017), although many correlational studies have linked norms to vaccination, no field studies have evaluated the use of descriptive norms to modify vaccination behaviour (cf. Leight & Safran, 2019). There have also only been a few survey studies and laboratory experiments exploring descriptive norms as drivers of vaccination (e.g., Hershey, Asch, Thumasathit, Meszaros, & Waters, 1994; Romley, Goutam, & Sood, 2016).

In this study, we aim to expand the literature by experimentally manipulating three descriptive‐norm levels (high vaccination coverage vs. low vaccination coverage vs. no coverage information communicated) and by assessing their influence on vaccination intentions.

Hypothesis 2 Exposure to descriptive social norms about vaccination (the level of vaccination coverage in one’s country) will influence vaccination intentions. Compared to participants who receive no information about the coverage, participants who are informed about high coverage will show higher vaccination intentions (Hypothesis 2a), whereas participants who are informed about low coverage will show lower intentions (Hypothesis 2b). Participants who are informed about high coverage will show higher intentions compared to participants who are informed about low coverage (Hypothesis 2c).

Although high descriptive‐norm messages have the potential to increase vaccination uptake, they can also promote a ‘backfire effect’. Employing both interactive games (e.g., Böhm, Betsch, & Korn, 2016; Ibuka, Li, Vietri, Chapman, & Galvani, 2014; Korn, Betsch, Böhm, & Meier, 2017) and hypothetical scenarios (Betsch et al., 2017; Vietri, Li, Galvani, & Chapman, 2011), previous studies have shown that learning about a high vaccine uptake prompts the individual to strategically ‘free‐ride’ on others’ protection and to refuse vaccination. This way, the ‘free‐rider’ also avoids some individual costs (e.g., money, time, inconvenience, vaccine side effects; Fine et al., 2011).

We did not expect the high descriptive norm in our study to decrease vaccination intentions in such a way. As will be detailed below, prior to learning about the descriptive norm, all of the participants learned about the social benefit of their own vaccination decision. It has been hypothesised that this framing of herd immunity activates an individual’s prosocial or other‐regarding preferences, thus preventing free‐riding (Betsch, Böhm, & Korn, 2013).

### Herd‐immunity threshold communication

This study also explored how communicating the vaccination coverage required to reach the herd‐immunity threshold influences vaccination intentions. Goal‐setting has been shown to be an effective strategy for behaviour change across a variety of domains, especially if the goal is set as a group goal, rather than an individual one (for a meta‐analysis, see Epton, Currie, & Armitage, 2017). In the context of vaccination behaviour, the collectively optimal group goal is the herd‐immunity threshold—that is, the proportion of the population that must be immunised to stop the infection from spreading and protect everyone (Fine et al., 2011).

In an interactive game, symbolically rewarding the attainment of a collectively optimal vaccination coverage positively affected uptake (Korn, Betsch, Böhm, & Meier, 2018). More closely related to this topic, Logan et al. (2018) presented a convenience sample of participants with the herd‐immunity threshold together with the definition of herd immunity and the actual community coverage from the previous year. This multi‐faceted intervention increased plans to get vaccinated against the flu the following year, but only among those who were not already knowledgeable about herd immunity.

Hypothesis 3 Participants who are informed about the numeric value of the herd‐immunity threshold will show higher vaccination intentions compared to the participants who are not informed about this value.

## Method

The approved Stage 1 protocol is available at: https://osf.io/jpku3.

### Study design

We ran an online experiment with a 2 (herd‐immunity explanation: present vs. absent) × 3 (descriptive norm: high vs. low vs. absent) × 2 (herd‐immunity threshold: present vs. absent) between‐subjects fractional design with seven groups (Table 1). Group 7 was the control which did not receive any experimental intervention to serve as a benchmark for the effect of herd‐immunity communication.

**Table 1:** Study design with factors, groups, and obtained sample sizes

	Factor 1	Factor 2	Factor 3	*n*
	Herd‐immunity explanation	Descriptive norm	Herd‐immunity threshold	
Levels	Present, absent	Low, high, absent	Present, absent	
Manipulation	Between‐subjects	Between‐subjects	Between‐subjects	
Group 1	Present	High	Present	45
Group 2	Present	Low	Present	45
Group 3	Present	High	Absent	45
Group 4	Present	Low	Absent	45
Group 5	Present	Absent	Present	46
Group 6	Present	Absent	Absent	45
Group 7	Absent	Absent	Absent	272
Total				543

The study used simple randomisation. The first randomisation (1:1) served to allocate half of the participants to the control group and the other half to the rest of the groups. In the second randomisation (1:1:1:1:1:1), the participants who had not been recruited to the control group were allocated to one of the six experimental groups. Participants did not know the group to which they had been allocated and researchers were blind to the group allocation process.

### Sampling plan

All participants had to meet the following inclusion criteria: (1) currently residing in the United Kingdom, (2) aged between 18 and 64 years, and (3) being confident in their English skills. Typically, individuals aged 65 or above are more susceptible to vaccine‐preventable diseases, which can be more severe than for younger people. Additionally, vaccines are less protective in older adults (Goldstein, 2012). It is possible that the community‐wide benefit emphasised in the herd‐immunity explanation would act as an incentive for younger adults to voluntarily get vaccinated to prevent illness among older adults (Chapman et al., 2012). Social‐benefit messaging, however, may not be effective among the elderly and otherwise vulnerable groups (Isler, Isler, Kopsacheilis, & Ferguson, 2020). Due to potential differential effects of herd‐immunity communication interventions associated with age, recruiting adults below 65 makes the findings of our study more directly comparable with the findings of the original study, which recruited participants from the same age group of the general population (Betsch et al., 2017).

Participants were recruited through advertisements on social media (e.g., Facebook groups, Twitter, Reddit), websites, and forums. To minimise self‐selection, the advertisements and informed consent page did not suggest that the study was related to vaccination. Participation was not compensated.

Data for this study were collected at the time of the COVID‐19 pandemic, between October 5 and 24 November 2020. The second half of the data collection period encompassed the second national lockdown (GOV.UK, 2020) but ended before COVID‐19 vaccinations were first rolled out in the United Kingdom (BBC News, 2020).

### Power analyses

We decided that the sample should be powered to detect the smallest effect of herd‐immunity communication that was plausible given previous research. Analysing the raw data from the original study (Betsch, Böhm, Korn, & Holtmann, 2017), we estimated the size of the effect at Partial Eta‐Squared (η^2^) = 0.024, across all locations. Three subsamples were large enough to allow for country‐level analysis; the effect remained small to medium in the United States and Germany (η^2^ = 0.049 and η^2^ = 0.073, respectively), but was small (η^2^ = 0.002) and did not reach statistical significance in South Korea (Cohen, 1988). The effect of communicating the social benefit of herd immunity was replicated by Betsch and Böhm (2018) among a sample of US parents; the effect sizes in the two experiments were η^2^ = 0.042 and η^2^ = 0.044. The target sample size is based on an *a priori* one‐way ANOVA power analysis using the R package {easypower} (McGarvey, 2015). Assuming α = .05, *N* = 531 suffices to detect the original effect size of 0.024 with .95 power. Target subsamples for experimental groups 1 through 6 was, therefore, *n* = 45; target subsample for the control group was *n* = 270. The total target sample was, thus, *N* = 540 participants.

We additionally conducted a sensitivity two‐way ANOVA power analysis for Hypotheses 2 and 3 using G*Power 3.1.9.4 software (Faul, Erdfelder, Lang, & Buchner, 2007). With the total sample size set at *n* = 270, α at .05, power at .95, the numerator degrees of freedom (*df*) at 2, and the number of groups at 6, our study would be able to detect a minimum effect size of η^2^ = 0.055 of the descriptive‐norm manipulation. With the numerator *df* set at 1 and the rest of the parameters remaining the same, it would be able to detect a minimum effect size of η^2^ = 0.046 of the herd‐immunity threshold manipulation. These effect sizes are small, but approaching the lower limit of what can be considered a moderate effect size, that is, η^2^ = 0.06 (Cohen, 1988).

The protocols of power analyses are available at https://osf.io/my2gf.

### Procedure and variables

The study was reviewed and approved by the Institutional Review Board at the University of Belgrade Department of Psychology (protocol #2019‐046). After informed consent, the questionnaire first assessed age, gender, country of residence, education, and socioeconomic status. After an attention check, participants received a textual explanation of herd immunity, accompanied by an animated infographic. Next, they were asked to imagine themselves in a scenario in which they had to decide whether to get vaccinated against a fictitious disease. The scenario informed participants about the disease and the vaccine, the herd‐immunity threshold, and the level of the vaccination coverage in their country. Following scenario‐recall questions, participants rated their intention to get vaccinated. Then, perceived riskiness of the infection and the disease were assessed. This was followed by a measure of vaccine hesitancy and a second attention check. Immediately after the experiment, all participants were fully debriefed and received a link to the WHO website on vaccinations for further information. It was emphasised again that all information regarding the disease and the vaccine was fictitious. The questionnaire is available at https://osf.io/hq9sv.

The online experiment was implemented in SoSci Survey. It was pre‐tested on a convenience sample of 14 people (two in each group) from the target population to ensure clarity and comprehension of the materials and fine‐tune the survey process. The data from the survey pre‐test were not included in the analyses.

### Manipulated variables

#### Herd‐immunity explanation

**Figure 1:**
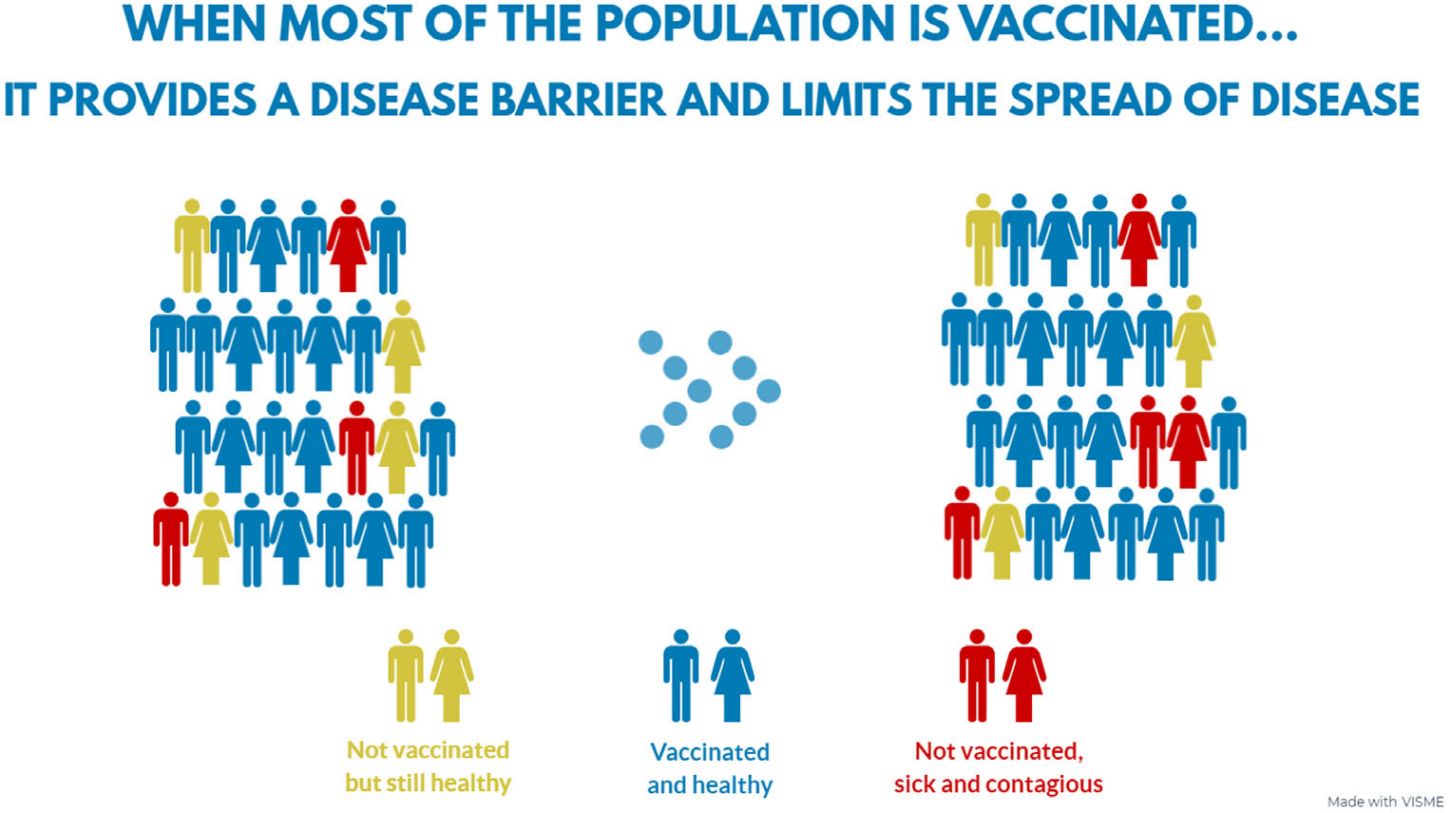
An example slide from the animated infographic. *Note*. This slide depicts the final outcome in the environment in which many people were vaccinated. The slide reads: ‘When most of the population is vaccinated … it provides a disease barrier and limits the spread of disease’. The legend shows three colours representing ‘not vaccinated but still healthy’, ‘vaccinated and healthy’, and ‘not vaccinated, sick and contagious’ individuals. All of the slides are available at https://osf.io/4hyjt. The animated infographic in full can be viewed at https://my.visme.co/projects/010jd830‐project‐animated.

Participants read a general explanation of herd immunity that emphasised the social benefit of getting vaccinated (i.e., protecting others in the community, especially the vulnerable). It did not feature the term ‘herd immunity’, but rather the term ‘community immunity’, and was 200 words long (see Appendix S1 for the full text). Participants also learned about herd immunity via a 40‐second animated infographic. It showed three environments with no vs. some vs. many people vaccinated and how the pathogen spreads in each one, infecting susceptible individuals (Figure 1). To prevent the participant from skipping the explanation and the infographic, the continue button was disabled for a specified minimum amount of time. If the participant reported any technical difficulties with starting the animation, they were shown a non‐animated infographic (depicting only the final outcome in the three environments). The control group received neither a text‐based nor an animated explanation of herd immunity. All of the materials have been developed by the authors.

#### Herd‐immunity threshold

Participants learned about the coverage needed to reach the herd‐immunity threshold for vaccination against a fictitious disease. To allow us to successfully manipulate the social norm, the threshold was set at 90%. To ease comprehension, the threshold was presented both as a percentage and as a number out of 10 (‘at least 9 out of 10 (90% of) people in a population need to get vaccinated to completely stop the [name of the disease] disease from spreading and to protect everyone’).

#### Descriptive social norm

Participants were given fictitious information about vaccination coverage in their country. To ease comprehension, this was presented both as a percentage and as a number out of 10 (e.g., ‘8 out of 10 (80% of) people in the United Kingdom have taken the vaccine’). The low coverage was set at 20% and the high coverage at 80%. It was important for these values to be extreme so that they were salient in an individual’s attention (Cialdini et al., 1990) and so that the range was wide enough for any reaction to herd behaviour to manifest itself.

### Outcome variable

All participants were faced with a vaccination decision task, which informed them about a severe fictitious disease and a fictitious vaccine. The use of fictitious materials excludes potential confounding variables, such as real infections and vaccine side effects experienced or observed by an individual (e.g., Chapman & Coups, 2006; Lane, MacDonald, Marti, & Dumolard, 2018). Additionally, it allows unconstrained manipulation of descriptive‐norm and herd‐immunity threshold levels. Participants first learned about the name of the virus and the path of infection (smear infection). Following Connolly and Reb (2003), the symptoms of the infection and vaccine side effects were described as equally likely (appearing in a small number of cases) and as very similar in content to ensure equal perceived riskiness. The vaccine was described as being easily available at no out‐of‐pocket cost and as 100% effective against infection with the disease. The source of information was not disclosed, as mistrust in healthcare authorities, government, and pharmaceutical companies have been shown to affect vaccine acceptance (Yaqub, Castle‐Clarke, Sevdalis, & Chataway, 2014). *Vaccination intention* was assessed by asking participants ‘If you had the opportunity to get vaccinated against [name of the disease] immediately, what would you do?’, on a 7‐point scale ranging from 1 = *I would definitely not get vaccinated* to 7 = *I would definitely get vaccinated*.

### Other measured variables

#### Sociodemographic variables

##### Age

Participants noted their age in years in an open‐response box.

##### Gender

Participants selected ‘female’, ‘male’, ‘non‐binary/third gender’, ‘prefer to self‐describe:’ or ‘prefer not to say’ to indicate their gender (Human Rights Campaign Guidelines).

##### Education

Participants reported their educational attainment in response to a single item (‘What is the highest educational level that you have attained?’). The response scale was adapted for the UK based on the International Standard Classification of Education.

##### Subjective socioeconomic status (SES)

Participants used a ladder with 10 steps to indicate their standing in the country relative to other people (Adler et al., 1994).

#### Vaccine hesitancy

Participants completed the five‐item version of the 5C scale of vaccine hesitancy (Betsch et al., 2018). Additionally, they answered a question about the compatibility of vaccines with their religious beliefs (Larson et al., 2016). All items appeared in a randomised order for each participant and were answered on a 7‐point scale ranging from 1 = *strongly disagree* to 7 = *strongly agree*. All items were recoded to reflect higher vaccine hesitancy (higher complacency, constraints, and calculation, and lower confidence, collective responsibility, and compatibility with religious beliefs). Since the internal consistency of the scale was lower than .70 (Cronbach’s alpha = .66), we conducted a sensitivity analysis. This indicated that the item assessing calculation (‘When I think about getting vaccinated, I weigh benefits and risks to make the best decision possible’) had extremely low corrected item‐total correlation (*r* =.071) and that removing it would improve reliability. We took the mean of the remaining five items to create a single measure of ‘vaccine hesitancy’ (Cronbach’s alpha = .72).

#### Perceived riskiness

Participants rated the perceived riskiness both of the infection (‘How risky do you judge a [name of the disease] infection to be if you do not get vaccinated?’) and the vaccine (‘How risky do you judge the vaccination against [name of the disease] to be?’), on a 0–100 slider (later transformed into a 1–101 scale). The questions were presented in a randomised order for each participant. To assess whether the disease and the vaccine were perceived as equally risky or not, we ran a paired t‐test on the perceived riskiness ratings.

### Analysis plan

We used R 4.0.5 (R Core Team, 2021) with {car} (Fox & Weisberg, 2019), {DescTools} (Signorell, 2021), {multcomp} (Hothorn, Bretz, & Westfall, 2008), {psych} (Revelle, 2020), and raincloud plots (Allen et al., 2021). Data and code are available at https://osf.io/zb7s3.

#### Hypotheses testing

In all of the following analyses, the dependent variable (DV) is ‘vaccination intention’. To test Hypothesis 1, we used a one‐way between‐subjects ANOVA. The independent variable (IV) is ‘herd‐immunity explanation’ (groups 1–6 vs. control). Using a one‐way between‐subjects ANOVA, we conducted an additional analysis only with those experimental groups, which more closely resemble the setting in the original study (Betsch et al., 2017), that is, only with the groups where herd‐immunity threshold is not communicated (groups 3, 4, and 6 vs. control). A successful replication of the herd‐immunity communication effect is defined as finding a statistically significant effect in the same *direction* as the original study.

To test Hypothesis 2 and Hypothesis 3, we used two‐way between‐subjects ANOVA without the interaction term. The IVs are ‘descriptive norm’ and ‘herd‐immunity threshold’. To test Hypotheses 2a, 2b, and 2c, we additionally performed pairwise comparisons between the three ‘descriptive norm’ levels.

We repeated all of the above analyses while controlling for age, gender, education, and socioeconomic status (ANCOVA with sociodemographic variables as covariates).

We applied the standard *p* < .05 level for determining if the ANOVA and pairwise comparisons tests suggest that the results are significantly different from those expected if the null hypothesis were correct. The post‐hoc Tukey’s tests adjust for multiple comparisons.

#### Exploratory analyses

To explore the interaction between the ‘descriptive norm’ (IV1) and the ‘herd‐immunity threshold’ (IV2), we performed a two‐way between‐subjects ANOVA with the interaction term, with ‘vaccination intention’ as the DV. We additionally tested the interaction between ‘vaccine hesitancy’ and the three factors (‘herd‐immunity explanation’, ‘descriptive norm’, ‘herd‐immunity threshold’) in the linear model, with the same DV.

#### Data exclusion

To ensure data quality, we included a recall test and attention checks. After participants received information regarding the descriptive norm and/or the herd‐immunity threshold, the recall test ensured they paid attention and remembered the values in their scenario. Depending on the group, the test offered one or two questions, with three choices (correct value, bogus value, ‘not sure’). In case of a failed recall, the scenario was presented up to two more times. Only those participants who passed the recall test were able to proceed with the experiment. Additionally, there were two attention‐check questions, asking participants to choose a specific response option (Berinsky, Margolis, & Sances, 2014). Participants who failed both attention checks were excluded from the analyses.

#### Missing data

Responses to all questions were mandatory to reduce data errors and omissions. However, education and socioeconomic status questions offered a ‘prefer not to say’ option (0 out of 549) and responses other than ‘female’ or ‘male’ were recoded as a missing value (19 out of 549). In analyses with the gender variable, pairwise deletion on missing data was done.

## Results

### Sample characteristics

The survey took participants approximately 7 minutes. Out of 549 participants who completed the study, six were excluded due to failed attention checks. The distribution of the remaining *N* = 543 participants by experimental group is shown in Table 1. Only 9 out of 271 participants reported technical difficulties and saw the non‐animated infographic.

As presented in Table 2, the majority of participants were female (67.77%) and had some higher education experience (75.51%). The mean vaccine hesitancy was low (2.1), with the distribution of responses being positively skewed (Shapiro‐Wilk test, *W*(543) = 0.88, *p* < .001).

**Table 2:** Sample characteristics (*N* = 543)

	*n* (%)	Range
Age in years (mean; *SD*)	38.0 (12.3)	18–64
Gender		
Female	368 (67.77)	
Male	157 (28.91)	
Non‐binary/third gender	10 (1.84)	
Prefer to self‐describe	2 (0.37)	
Prefer not to say	6 (1.10)	
Education		
No formal education	6 (1.10)	
Completed secondary school	48 (8.84)	
Completed post‐16 education	79 (14.55)	
Some higher education	82 (15.10)	
Completed higher education	177 (32.60)	
Completed advanced degree	151 (27.81)	
Subjective socioeconomic status (mean; *SD*)	5.5 (1.7)	1–10
Vaccine hesitancy (mean; *SD*)	2.1 (1.1)	1–7
Perceived riskiness (mean; *SD*)^a^		
Riskiness of the infection	56.1 (30.4)	1–101
Riskiness of the vaccine	30.4 (28.3)	1–101

Note

*SD* = standard deviation.

^a^ The riskiness of the infection with the disease was perceived as statically significantly higher than the riskiness of taking the vaccine, *t*(542) = 14.46, *p* < .001.

### Hypotheses testing

**Figure 2:**
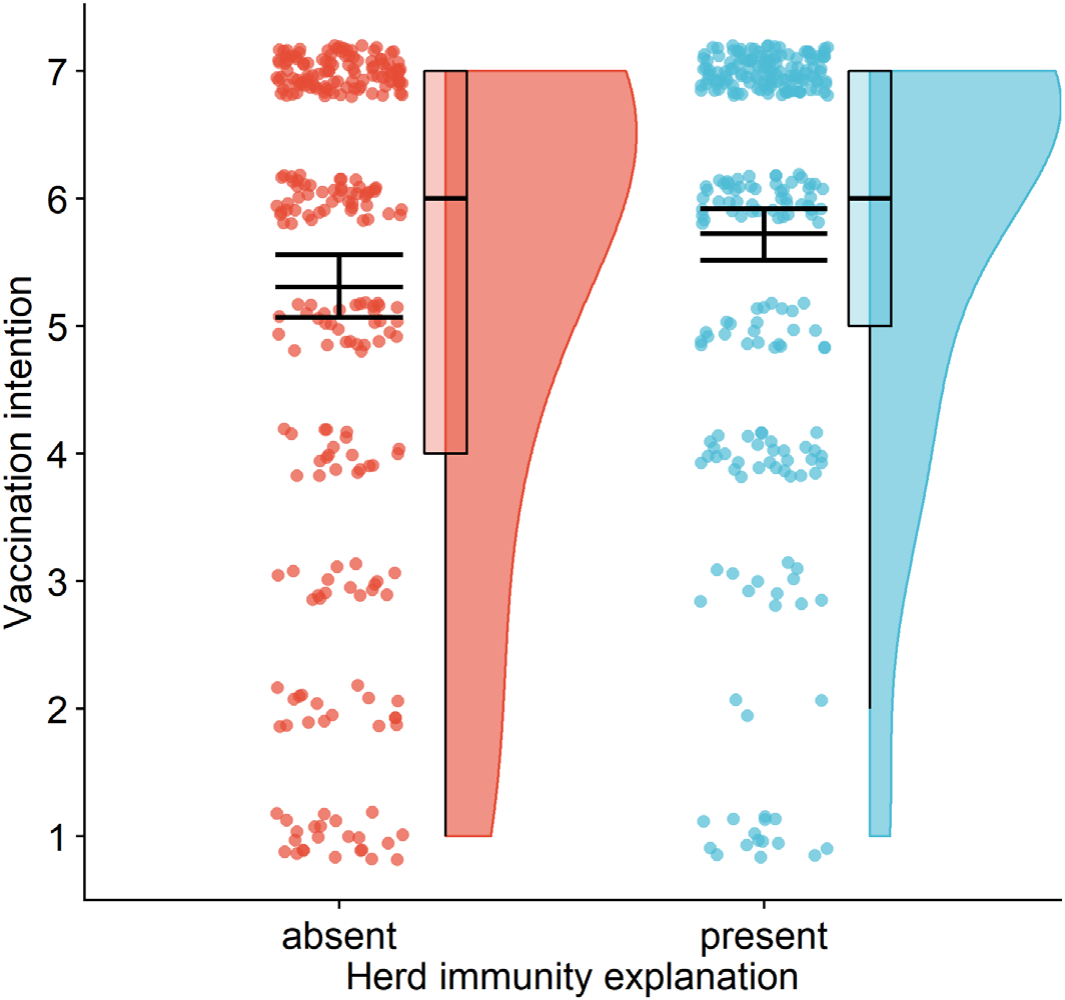
Vaccination intentions depending on whether herd‐immunity explanation was provided. *Note*. Communicating herd immunity via text and animated infographic was effective in increasing vaccination intentions. The figure shows a raincloud plot with the distribution of the data and jittered raw data; the box plot indicates the interquartile range from the 25th to the 75th percentile, including the median; the mean (with 95% confidence interval) is plotted on top of the jittered points.

Communicating herd immunity significantly increased vaccination intentions compared to the control (*M* = 5.7, *SD* = 1.7 vs. *M* = 5.3, *SD* = 2.0), *F*(1,541) = 6.97, *p* = .009, η^2^ = 0.013 (Figure 2), supporting Hypothesis 1. The effect remained significant after controlling for sociodemographic variables, *F*(1,519) = 5.92, *p* = .018, η^2^ = 0.011. After excluding the groups where the herd‐immunity threshold was communicated (and without any covariates included in the model), the effect was no longer significant, although it remained in the same direction, *F*(1,405) = 3.48, *p* = .063, η^2^ = 0.009.

**Figure 3:**
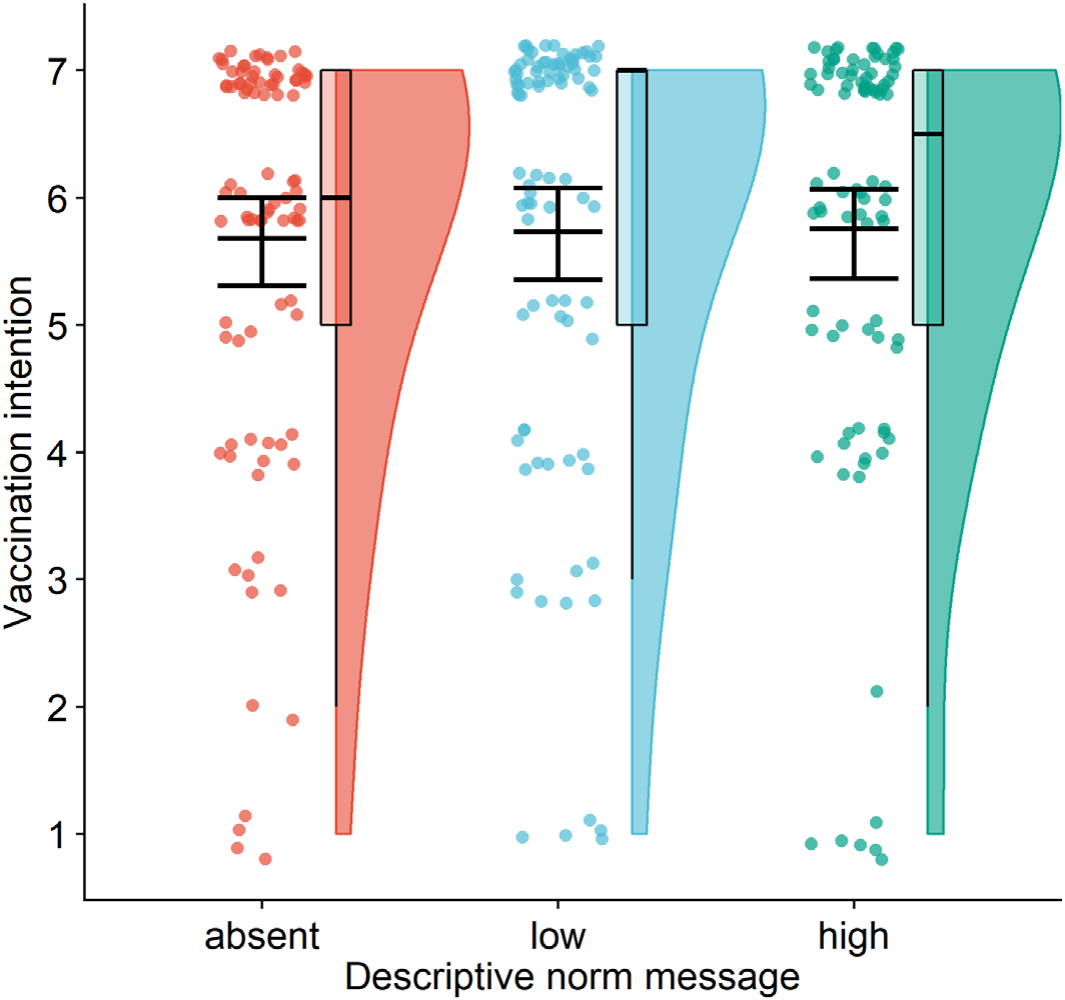
Vaccination intentions depending on the levels of the descriptive norm message. *Note*. Communicating different descriptive norm messages (no‐coverage vs. low‐coverage [20%] vs. high‐coverage [80%] message) alongside herd immunity was not effective in increasing vaccination intentions. The figure shows a raincloud plot with the distribution of the data and jittered raw data; the box plot indicates the interquartile range from the 25th to the 75th percentile, including the median; the mean (with 95% confidence interval) is plotted on top of the jittered points.

Exposure to descriptive norms did not influence vaccination intentions, *F*(2,267) = 0.05, *p* = .956, η^2^ < 0.001, not supporting Hypothesis 2 (Figure 3). Neither low (*M* = 5.7, *SD* = 1.8) nor high norms (*M* = 5.8, *SD* = 1.7) were significantly different from the no‐coverage message (*M* = 5.7, *SD* = 1.7) (estimate = 0.05, *SE* = 0.26, *p* = .977, 95% CI [−0.55, 0.66] and estimate = 0.07, *SE* = 0.26, *p* = .954, 95% CI [−0.53, 0.68], respectively). There was also no difference between low and high norms, estimate = −0.02, *SE* = 0.26, *p* = .996, 95% CI [−0.63, 0.58]. Hypotheses 2a, 2b, and 2c were, therefore, not supported. The main effect of norms did not change after controlling for sociodemographic variables (*F*(2,255) = 0.05, *p* = .951, η^2^ < 0.001), and neither did the differences between the levels.

**Figure 4:**
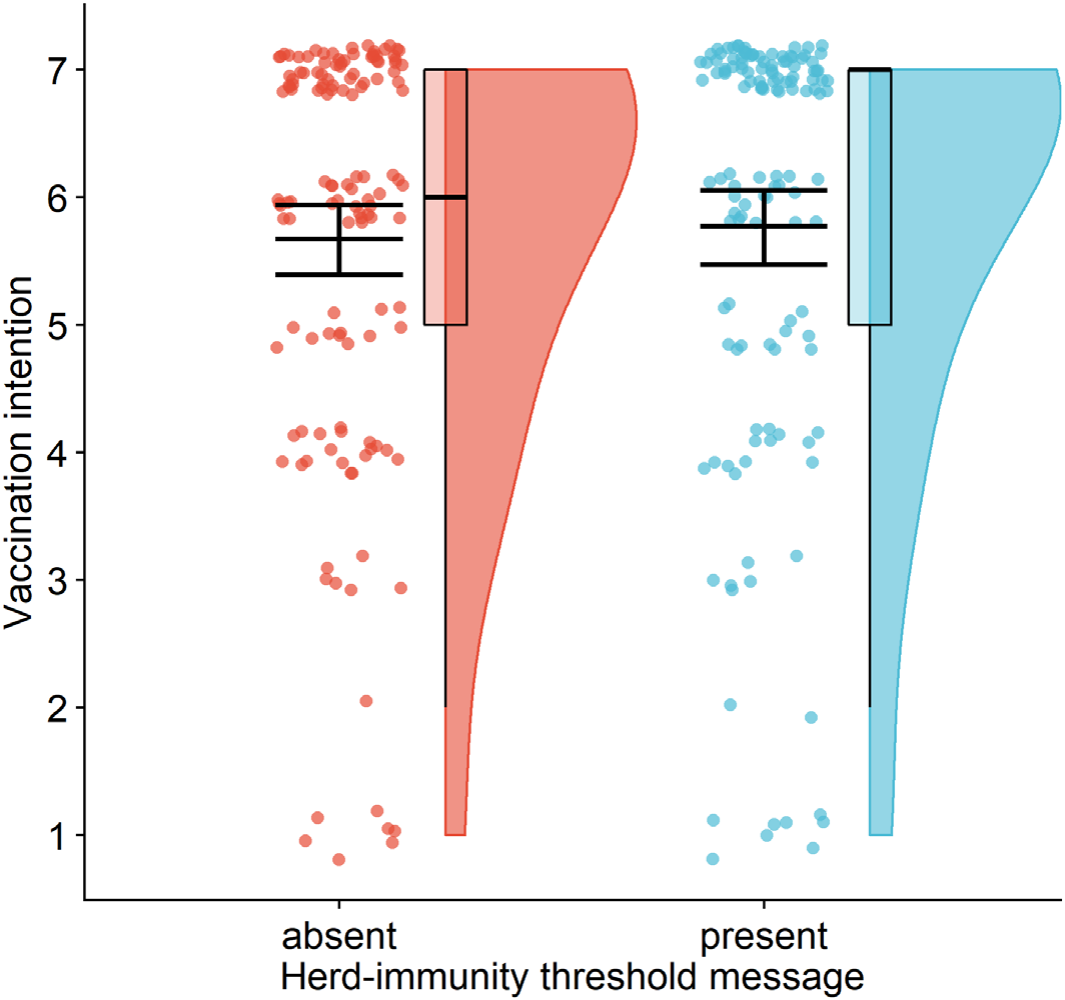
Vaccination intentions depending on whether the herd‐immunity threshold was provided. *Note*. Communicating the herd‐immunity threshold alongside herd immunity was not effective in increasing vaccination intentions. The figure shows a raincloud plot with the distribution of the data and jittered raw data; the box plot indicates the interquartile range from the 25th to the 75th percentile, including the median; the mean (with 95% confidence interval) is plotted on top of the jittered points.

The presence of the herd‐immunity threshold did not influence vaccination intentions, *F*(1,267) = 0.22, *p* = .639, η^2^ = 0.001, not supporting Hypothesis 3 (Figure 4). Intentions of the participants who were informed about the threshold (*M* = 5.8, *SD* = 1.8) were not significantly different from the intentions of the participants who were not informed about it (*M* = 5.7, *SD* = 1.7). This effect did not change after controlling for sociodemographic variables, *F*(1,255) = 0.45, *p* = .501, η^2^ = 0.002.

### Exploratory analyses

We detected no significant interaction between the descriptive‐norm and herd‐immunity threshold factors, *F*(2,265) = 1.32, *p* = .269, η^2^ = 0.010. When the threshold information was absent, mean vaccination intentions were 5.7 (*SD* = 1.6), 5.4 (*SD* = 1.8), and 5.8 (*SD* = 1.7) for the no‐norm, low‐norm, and high‐norm level, respectively. When the threshold information was present, mean vaccination intentions were 5.6 (*SD* = 1.8), 6.0 (*SD* = 1.7), and 5.7 (*SD* = 1.8), for the no‐norm, low‐norm, and high‐norm level, respectively.

We detected no significant interaction between vaccine hesitancy and either of the three factors (herd‐immunity explanation, *F*(1,539) = 0.51, *p* = .476, η^2^ = 0.001; descriptive norm, *F*(2,265) = 1.72, *p* = .181, η^2^ = 0.013; herd‐immunity threshold, *F*(1,267) = 0.55, *p* = .460, η^2^ = 0.002). We thus did not proceed with testing the moderating effect of vaccine hesitancy on the relation between the three factors and vaccination intentions.

## Discussion

This Registered Report successfully replicated Betsch et al.’s (2017) finding that communicating the social benefits of herd immunity increases stated vaccination intentions against a fictitious disease, with novel materials—a differently worded explanation and an animated infographic—and with participants from another country—the United Kingdom. Communicating the descriptive norm (low or high vaccination coverage in the country) or the threshold (coverage needed to stop disease transmission) alongside herd immunity demonstrated no observable effect. It is possible that norms and the threshold showed no effect precisely because all participants were familiarised with the concept of herd immunity. Future studies should further disentangle the relation between these three factors.

When it comes to herd immunity, the observed effect size (Partial Eta‐Squared = 0.013 or Cohen’s *d* = 0.23) was smaller than in previous studies (e.g., Betsch & Böhm, 2018; Betsch et al., 2017). This might be due to the pandemic context in which participants had been living. Firstly, some preventative measures (such as physical distancing or mask wearing) required people to bear a personal cost to benefit others or society as a whole (for a review, see Capraro et al., forthcoming). This might have caused participants to have a generally stronger focus on social benefits, which might have consequently reduced the observed herd‐immunity effect. Secondly, in March 2020, herd immunity briefly came to be seen as the UK government’s strategy to respond to COVID‐19, attracting heavy criticism and public backlash. The confusion stemmed from interviews in which government advisers appeared to suggest that one way to manage the epidemic would be to naturally reach herd immunity by aiming for 60% of the population to fall ill (e.g., Freedman, 2020; Sasse, Haddon, & Nice, 2020; Yong, 2020). Although our study materials mentioned the term ‘community immunity’ only, explaining that it was generated through vaccination (not infection), some participants might have misinterpreted the materials or felt repelled by them due to confusing public messaging earlier that year.

In the context of the COVID‐19 pandemic, some recent self‐reported online surveys pointed to the usefulness of social‐benefit messaging in promoting vaccine acceptance (in France, Schwarzinger, Watson, Arwidson, Alla, & Luchini, 2021; in the United Kingdom, Pfattheicher et al., in press). However, data from a representative UK sample did not corroborate these findings (Freeman et al., 2021). In this study, message type had no effect for people willing to be vaccinated and people who were doubtful. However, highlighting *individual* benefits increased vaccination intentions in people who were strongly hesitant, more than highlighting collective benefits of not getting ill and not transmitting the virus. This study also provided preliminary findings suggesting that ethnicity might moderate the impact of different messages on COVID‐19 vaccine hesitancy (Freeman et al., 2021). The effectiveness of herd‐immunity appeals is also likely contingent on the scientific consensus on whether COVID‐19 vaccines provide herd immunity in the first place and on people's knowledge on this issue (Korn, Böhm, & Betsch, 2021).

More research is also needed to uncover how best to apply existing theories on descriptive‐norm communication and collective‐goal setting. Future studies could focus on testing more realistic interventions of using normative messages with factual information about others’ vaccine intentions or behaviours that correct people’s underestimation of how many other people accept a vaccine (see, for example, Moehring et al., 2021).

The effect of communicating the herd‐immunity threshold at different levels of vaccination coverage should be further explored in studies adequately powered to detect a potential interaction effect. One question of practical relevance would be whether public communication should highlight the threshold value when the coverage is very close or very far from reaching it. In the context of collective goals, some studies suggest that people would be more likely to contribute as a goal nears completion, in part because this provides them with a heightened sense that their action will have an impact (e.g., Cryder, Loewenstein, & Seltman, 2013; Moussaoui & Desrichard, 2017; see also Anik & Norton, 2020).

The main limitation of this study was that the sample was not representative of the UK population. The results, therefore, cannot be presumed to generalise to the whole population. In particular, most of the participants were highly educated and reported, on average, low vaccine hesitancy. Another limitation is that ethnicity was not recorded. It is possible that people who are strongly hesitant or come from subgroups with low vaccination acceptance would react less favourably to social‐benefit messaging (e.g., Freeman et al., 2021). To develop more tailored, culturally sensitive communication strategies, future studies should explore intersections of social categories and issues that make people more likely to refuse vaccination (Independent Scientific Advisory Group for Emergencies, 2021).

This study explored three intervention strategies that leverage social processes to motivate vaccination—herd immunity, the herd‐immunity threshold, and descriptive norms—with a sample of non‐senior adults residing in the United Kingdom. We conceptually replicated a previous finding that communicating the social benefit of herd immunity increases stated vaccination intentions. To provide further empirical guidance for effective and scalable communication strategies that rely on social nudges, it might be useful to replicate this study design with real‐world vaccine‐preventable diseases; to conduct the studies in other countries and with samples that are representative of the population (also with respect to vaccine hesitancy); and to assess the long‐term effects of providing people with information about herd behaviour.

## Conflicts of interest

All authors declare no conflict of interest.

## Author Contributions

Aleksandra Lazić (Conceptualization; Data curation; Formal analysis; Investigation; Methodology; Project administration; Supervision; Validation; Visualization; Writing – original draft; Writing – review & editing); Kalina Nikolova Kalinova (Conceptualization; Investigation; Methodology; Writing – review & editing); Jali Packer (Conceptualization; Investigation; Methodology; Writing – review & editing); Riinu Pae (Conceptualization; Investigation; Methodology; Writing – review & editing); Marija B. Petrović (Conceptualization; Formal analysis; Investigation; Methodology; Writing – review & editing); Dora Popović (Conceptualization; Investigation; Methodology; Writing – review & editing); D. Elisabeth C. Sievert (Conceptualization; Investigation; Methodology; Project administration; Writing – review & editing); Natalie Stafford‐Johnson (Conceptualization; Investigation; Methodology; Writing – review & editing).

## Supporting information


**Appendix S1**. Herd immunity explanation.

## Data Availability

The approved Stage 1 protocol is available at: https://osf.io/jpku3. The materials, data, and code that support the findings of this study are made openly available in the Open Science Framework at: https://osf.io/zb7s3.
