# Supplementary material for: Social nudges for vaccination: How communicating herd behaviour influences vaccination intentions
Source: Br J Health Psychol. 2021 Sep 8;26(4):1219–37. doi: 10.1111/bjhp.12556 (PMC8646271; doi:10.1111/bjhp.12556)
Supplement: Supplementary file 1 — Appendix S1. Herd immunity explanation. [file BJHP-26--s001.docx]

**Appendix S1**

**Herd Immunity Explanation**

**What Is Community Immunity?**

Getting vaccinated protects us in two ways. It protects the individual against an infectious disease. Not only does the vaccine stop the individual from catching the disease, it also means that they can’t pass it on to other people in their community. So, vaccines don’t only protect you but also those around you.

Germs can spread quickly through communities and make a lot of people sick. However, as more people get vaccinated, it is harder for the disease to spread. When a high enough number of people in the community are vaccinated, the disease can be wiped out altogether.

**Who Does Community Immunity Protect?**

Community immunity protects everyone but it’s especially important for individuals who are more vulnerable to infectious diseases.

When you get vaccinated, you protect people who can't get vaccinated - such as newborn babies, older people, and people with weak immune systems (like people who have cancer, HIV/AIDS, type 1 diabetes, or other health conditions).

These people rely on community immunity to protect them. So, even if you don’t feel personally at risk from a disease, getting yourself vaccinated benefits others in your family and your community.

Getting vaccinated protects you and those around you.
